# Pompe disease: a country-wide molecular screening in a cohort of 15,068 study participants

**DOI:** 10.3389/fmolb.2025.1745925

**Published:** 2026-01-29

**Authors:** Aleksander Pushkov, Daria Chudakova, Ilya Zhanin, Sergey Nikitin, Elena Basargina, Lydmila Kuzenkova, Daria Andreeva, Maria Vasil’eva, Sergey Vasichkin, Albina Gabitova, Elena Gaisina, Elena Saifullina, Amina Gamzatova, Olga Gilvanova, Vyacheslav Dudin, Olga Ergina, Sergey Kurbatov, Elena Noskova, Elena Osipova, Olga Tihonova, Ekaterina Fedotova, Evgeny Nuzhny, Viktoria Chernikova, Anastasia Yamshchikova, Lubov Kyzina, Natalia Irinina, Olga Rohlenko, Yulia Kutkova, Natalia Popkova, Alsy Ahunova, Alina Alexeeva, Natalia Mazanova, Nataliya Sdvigova, Leyla Gandaeva, Olga Zharova, Tatyana Podkletnova, Alexey Sukhozhenko, Anastasia Rusakova, Dmitry Demianov, Aleksandr Pakhomov, Andrey Fisenko, Kirill Savostyanov

**Affiliations:** 1 National Medical Research Center of Children’s Health of the Ministry of Health of the Russian Federation Moscow Russia, Moscow, Russia; 2 Research Centre for Medical Genetics, Moscow, Russia; 3 Children’s City Clinical Hospital №11 Yekaterinburg Russia, Yekaterinburg, Russia; 4 Medical and Genetic Center of the State Budget Healthcare Institution Clinical Center for Family Health and Reproduction, Novosibirsk, Russia; 5 Central Clinical Hospital with a Polyclinic of the Administration of the President of the Russian Federation, Moscow, Russia; 6 Republican Medical Genetic Center, Ufa, Russia; 7 Republican Perinatal Center named after Omarov S.-M.A, Makhachkala, Russia; 8 Moscow Clinical Scientific and Practical Center named after A. S. Loginov, Moscow, Russia; 9 Center for Cardiology and Neurology, Kirov, Russia; 10 Arkhangelsk Regional Clinical Hospital, Arkhangelsk, Russia; 11 Voronezh State Medical University named after N.N. Burdenko, Voronezh, Russia; 12 City Clinical Hospital No1, Chelyabinsk, Russia; 13 Regional Clinical Hospital, Tver, Russia; 14 Immanuel Kant Baltic Federal University, Kaliningrad, Russia; 15 Research Center of Neurology, Moscow, Russia; 16 Samara State Medical University, Samara, Russia; 17 Research Institute for Complex Problems of Hygiene and Occupational Diseases, Novokuznetsk, Russia; 18 Smagul Kaishibaev Neurology and Neurorehabilitation Institute, Almaty, Russia; 19 Regional Clinical Hospital, Vladimir, Russia; 20 Kuzbass Regional Clinical Hospital named after S.V. Belyaev, Kemerovo, Russia; 21 Ulyanovsk Regional Children’s Clinical Hospital named Yu.F. Goryachev, Ulyanovsk, Russia; 22 Perinatal Center, Smolensk, Russia; 23 Republican Clinical Hospital, Kazan, Russia; 24 Federal Research Center “Fundamentals of Biotechnology” of the Russian Academy of Sciences, Moscow, Russia

**Keywords:** gene GAA, genetic screening, infantile-onset pompe disease (IOPD), late-onset pompe disease (LOPD), next-generation sequencing, NGS, pompe disease, rare hereditary diseases

## Abstract

**Introduction:**

Pompe disease (PD) is a rare inherited recessive autosomal disorder caused by pathogenic nucleotide variants within the gene *GAA*, encoding Acid alpha-glucosidase (GAA), the lysosomal enzyme catalyzing glycogen breakdown to glucose.

**Methods:**

We performed molecular screening in a cohort of 15,068 participants with suspected PD of a wide range of age from several regions of Russia. Two screenings had been undertaken from 2014 to 2025, and 2021 to 2025, respectively: 13,128 patients were screened using “two-tier one-gene” algorithm (measurement of GAA activity in dried blood spots followed by Sanger sequencing of the GAA) and 1940 patients were screened using Next-Generation Sequencing (NGS)-based algorithm (NGS of the panel of genes linked to neuromuscular disorders, *ANO5, CAPN3, DYSF, FKRP, SGCA, SGCB, SGCD, SGCG, TCAP*, and *GAA*, followed by measurement of GAA activity when needed).

**Results:**

63 causative nucleotide variants in the *GAA* gene were detected, the most common being c.-32-13T>G (28 patients) followed by c.2662G>T (10 patients). 25 novel nucleotide variants in the *GAA* gene were described. Of 1,940 patients screened using an NGS-based algorithm, 138 patients (7.1%) were diagnosed with various muscular dystrophies, myopathies, or PD. The majority of these individuals had biallelic variants in the *CAPN3* gene (30%), indicative of calpainopathy. Total 57 (0.47%) and 8 (0.41%) patients were diagnosed with PD as a result of “two-tier one-gene” or NGS-based algorithms, respectively.

**Discussion:**

Overall, our study is the first large-scale country-wide selective screening for PD in Russia based on sequencing and GAA activity measurement and providing the most comprehensive overview of genetics of PD in this study population.

## 1 Introduction

Pompe disease (PD, OMIM#232300), also known as Glycogen Storage Disease II (GSDII) or Acid Maltase deficiency, is a rare multisystem hereditary recessive autosomal disorder. It is caused by causative nucleotide variants (NVs) within the gene *GAA* (OMIM 606800) that is located on chromosome 17q25.2-q25.3 and encodes lysosomal enzyme Acid alpha-glucosidase (GAA, Enzyme Commission number 3.2.1.20, also known as Acid Maltase) (Reuser et al., 2019). Such NVs result in the absence or reduced enzymatic activity of GAA. The GAA is the only enzyme capable of catalyzing glycogen breakdown to glucose at acidic pH of lysosomal environment; thus, its inactivation or decreased activity causes aberrant accumulation of glycogen within lysosomes. In turn, it inevitably leads to lysosomal swelling and rupture, disturbed cytoplasmic glycogen metabolism, as well as changes in calcium homeostasis, mitochondrial dysfunction, disruption of lysosome-dependent signaling pathways (such as mTORC1/AMPK) and subsequent pathologies of autophagy (summarized in (Sánchez-Porras et al., 2023; Zhang et al., 2024) and others). All of this results in a continuum of phenotypes and a broad range of PD manifestations.

The predicted genetic prevalence of PD in the general population varies by population. In particular, it is extremely low in Finland (1:1,056,444) and relatively high in Non-Finnish European (1:13,756) and East Asian population (1:12,125), whereas in other populations it is lower than in Non-Finnish Europeans but higher than in Finnish; namely, for Ashkenazi Jews it is estimated as 1:22,851, for African/African-American 1:26,560, for Latino/Admixed American 1:57,620, and for South Asian 1:93,087 (Park, 2021).

PD can affect people of any age. Traditionally, it is classified into two subtypes - Infantile Onset PD (IOPD) occurring at birth or within the first 12 months of life, also known as classic form of PD, and Late Onset PD (LOPD) occurring after the first 12 months of life. Additionally, depending on the time of onset, PD can be subdivided into infantile, late-infantile, childhood, juvenile, or adult PD. IOPD is characterized by severe muscle weakness, hypertrophic cardiomyopathy and respiratory insufficiency. It is more detrimental compared to LOPD and if untreated usually results in death in the first 18 months of life. Common feature of LOPD is progressive dysfunction of skeletal muscle (“muscle weakness”) and subsequent respiratory failure. Classic IOPD is caused by complete functional deficiency of GAA defined as a less than ∼3 percent of activity, whereas levels of GAA activity in less severe late onset cases of PD are in the range of 3–30 percent of normal (van der Ploeg and Reuser, 2008). Some IOPD patients can also have an atypical (non-classic) phenotype, with manifestation by the end of the first year of life and symptoms less severe than in case of the classic one. At birth PD is usually asymptomatic. All of this complicates diagnostics and causes delays in providing appropriate therapy.

The gene *GAA* has been comprehensively characterized; hitherto, it is considered that it comprises 20 exons, non-coding exon 1 and coding exons 2–20. More than 1,000 sequence variants in the *GAA* gene can be found in the ClinVar database (https://www.ncbi.nlm.nih.gov/clinvar/, accessed on 2 October 2025). Of them, more than 540 nucleotide variants have been reported so far as linked to PD (the Pompe disease *GAA* variant database, https://www.pompevariantdatabase.nl/pompe_mutations_list.php?orderby=aMut_ID1, accessed on 2 October 2025), and the database was extended by linking disease-associated variants to clinical severity (Niño et al., 2019).

The Enzyme replacement therapy (ERT) - systematic intravenous infusion with a recombinant human GAA (rhGAA), that has been used in clinics for almost two decades allows to mitigate the GAA dysfunction, at least partially. Early ERT in IOPD may improve respiratory as well as some muscular functions and to some degree reverse cardiomyopathy, though with limited impact on skeletal muscles. There is a strong association of the ventilation-free survival and very early ERT initiation for IOPD patients (Kishnani et al., 2024).

Moreover, an immune response to rhGAA may render therapy ineffective. It’s been shown that ERT efficiency is determined at least in part by a so-called Cross-reactive immunologic material (CRIM) status (presence or absence of residual activity GAA). CRIM-negative infant PD patients (“GAA deficient”) usually have a worse clinical outcome on ERT compared to CRIM-positive infants, whose with dysfunctional or aberrant endogenous GAA, because of production of anti-rhGAA antibodies (anti-drug antibodies (ADA)) in CRIM-negative individuals (Gandaeva et al., 2022). However, it’s not always the case (Khallaf et al., 2012). Nevertheless, High-sustained rhGAA IgG antibody titers (HSAT) are observed in the majority of CRIM-negative and in some CRIM-positive individuals. In the case of CRIM-negative status, immunomodulatory therapy is recommended before or immediately after the initiation of ERT (Chen et al., 2024). CRIM-status can be determined by immunological approaches, such as Western blotting, but usually is accurately predicted based on the type of pathogenic nucleotide variants in the *GAA* gene (Bali et al., 2015).

In LOPD, an impact of ERT on skeletal muscle has been reported (Ripolone et al., 2018). It’s unknown whether ERT is needed or beneficial for asymptomatic individuals with LOPD (Bolano-Diaz and Diaz-Manera, 2022). However, some patients are less responsive to ERT and its efficiency varies in different tissues. Moreover, an immune response to rhGAA may render therapy ineffective. Hitherto, ERT is the only approved therapy of PD, apart from support therapies (physical therapy, artificial ventilation, etc). To overcome limitations of ERT discussed above, other curative approaches to PD are currently being developed, such as gene therapy introducing wild type *GAA* gene (comprehensively reviewed in (Unnisa et al., 2022)), pharmacological chaperone therapy (PCT) in case of dysfunctional GAA (comprehensively reviewed in (Borie-Guichot et al., 2021)), splicing modulation by antisense oligonucleotides (Aung-Htut et al., 2020) and others (Bellotti et al., 2020). For some of them, it’s of critical importance to determine disease-associated pathogenic nucleotide variants in the *GAA* gene of the patient.

Alarmingly, clinical presentation of LOPD is very broad and may overlap with other neuro-muscular conditions, leading to underdiagnoses of PD and delays in treatment. LOPD can be mistaken for Duchenne disease, Becker and limb-girdle types of muscular dystrophy, Myotonic dystrophy-2, Rigid spine syndrome, Myasthenia gravis, Spinal muscular atrophy, Polymyositis, Glycogen storage disease types IIIa, IV, V and VII, Danon disease, Mitochondrial myopathies, and Asymptomatic hyperCKemia (Barba-Romero et al., 2012). Even in the case of IOPD, which is characterized by rather uniform clinical features compared to LOPD, there is a spectrum of phenotypes and symptoms.

There is a significant diagnostic delay for PD compared to other more common conditions; for LOPD it can reach 12–480 (median 144) months, and reported delays in case of IOPD was 1.5–3 (median 2.5) months (Lagler et al., 2019). This is an unsatisfactory situation given the progressive nature of disease, severity of symptoms, and consequences of the therapy delay. An untreated PD is progressively debilitating and in the case of IOPD is life-threatening, thus it is of crucial importance to diagnose PD timely and precisely.

In some countries, PD is included in the recommended uniform screening program of heritable disorders in newborns and children (or other, equivalent programs) (Hale et al., 2020). In other regions, selective screening of high-risk population can be performed as an alternative to newborn screening (NS). Currently, no standard worldwide accepted protocol exists for the clinical molecular diagnosis of PD. The guidelines of European consensus for starting ERT recommend that the LOPD diagnosis is established if at least two disease-associated pathogenic nucleotide variants in the *GAA* gene are present and GAA deficiency is determined (van der Ploeg et al., 2017). This can be determined with the aid of a first-tier analysis via biochemical methods such as enzyme assays measuring GAA activity, usually using a dried blood spot (DBS) method (Goldstein et al., 2009), and *GAA* genotyping using various sequencing approaches, usually Sanger sequencing. Notably, some sequence variants within the *GAA* gene cause a decrease of the GAA enzyme activity, but do not cause PD - a pseudodeficiency phenomenon. The presence of pseudodeficiency variants might lead to false-positive results in enzyme assay-based tests for PD.

Next-Generation Sequencing (NGS) is a fast and relatively cost-effective high-throughput method that makes it possible to perform sequencing of multiple targets in a single reaction and test concurrently for several hereditary conditions. It’s a significant step forward compared to conventional long diagnostic journeys and “step-by-step” analysis confirming or ruling out certain diagnoses in a relatively time-consuming manner. Incorporation of NGS into clinical practice, subsequently, led to a paradigm shift in the algorithms of rare disease screening. A growing number of works report NGS as a preferable tool for PD diagnostics. Moreover, it’s been reported that deep NGS might correct diagnostic pitfalls of Sanger sequencing-based approach (Tsai et al., 2017).

Previously, NGS with targeted gene panels including *GAA* and other genes involved in neuromuscular diseases or glycogen storage disorders allowed to make a definite diagnosis of PD in several cohorts (Bevilacqua et al., 2020; Savarese et al., 2018; Lévesque et al., 2016). Moreover, it also allowed identifying novel sequence alterations within the *GAA* gene (King et al., 2023). The ongoing Lantern Project program applies EA of GAA as well as NGS, using focused multigene panel including *GAA* and other “neuromuscular” genes, to PD diagnostics (King et al., 2023), and as a result 140 study participants were diagnosed with PD during a period from 2018 till 2021. Importantly, several individuals from the cohort had sequence variants in both *GAA* and other genes from the panel. As we and others demonstrated recently, cases of coexistence of two or more rare hereditary diseases within individual patients (Burykina et al., 2025) advocate for comprehensive genetic testing. Indeed, having PD does not rule out the possibility of being affected by other diseases which have “neuromuscular” clinical presentations similar to PD.

Here, we present results of the country-wide selective screening in a cohort of 15,060 participants with suspected PD from several regions of Russia using two algorithms. First one is based on the measurement of GAA activity in dried blood spots (DBS) followed by Sanger sequencing of the *GAA* and second one uses Next-Generation Sequencing (NGS) of the panel of genes linked to neuromuscular disorders, followed by measurement of GAA activity in patients with biallelic variant in *GAA* gene.

The current study is the largest selective molecular screening for PD in the Russia so far, providing the most comprehensive overview of genetics of PD in this study population.

## 2 Materials and methods

### 2.1 Study cohort and participants recruitment

The patients with suspected PD have been recruited to this single-center study governed by the Federal State Autonomous Institute National Medical Research Center for Children’s Health of the Russian Federation Ministry of Health on an ongoing basis starting from 2014 till current. The data from 2014 to 2025 are presented in this study. The participants were recruited from several geographically distant regions of the Russian Federation. Criteria for inclusion into the cohort were, in case of suspected IOPD - pronounced diffuse muscle hypotonia, myopathy, cardiomegaly, cardiomyopathy, macroglossia, aberrant heart rate; and in the case of suspected LOPD - diffuse muscle hypotonia, muscle weakness, presence of “Gowers’ sign” when standing up, difficulty walking, increased serum creatine kinase (CK) level. Other neuromuscular symptoms warranting recruitment into the cohort for NGS-based screening were 1) muscular weakness of the lower limbs, abdominal trunk muscles, back muscles 2) elevated creatine phosphokinase (200–2000 u/l) 3) elevated ALT/AST ratio. We screened 1,940 patients (795 females and 1,145 males) using an NGS-based algorithm. The median age at screening was 30.3 years, with ages ranging from 7 months to 72 years. For two-tier algorithm number of patients was 12,128 (5,700 females and 6,428 males). The median age at screening was 17.6 years, with ages ranging from 1 month to 88 years. Informed consent was obtained from all subjects involved in the study or from their legal guardians, in accordance with the Helsinki declaration. The study was approved by the Ethics Committee of National Medical Research Center for Children’s Health, Moscow, Russian Federation.

### 2.2 GAA activity measurement in DBS

The peripheral blood stains, ∼3 ul, were dried on a filter pad paper (Whatman carta-filter No. 903) and dried blood spots (DBS) were processed using standard procedure, as described previously elsewhere. Briefly, GAA activity was measured by tandem mass spectrometry (MS/MS)-based method on “MaXis Impact” (Bruker Daltonics, Bremen, Germany) with positive ionization in electrospray. Ion detection was carried out using flow-injection analysis with the Multiple Reaction Monitoring. The mixture of acetonitrile and water with the addition of formic acid was used as an eluent. The ion detection was in the range 100–550 m/z with an accuracy of determining masses of at least 5 ppm and solution of at least 20,000 (FWHM). The analysis time for one sample was 3 min. The data were analyzed using the built-in package software “Bruker Data Analysis 4.1”. In the case of low activity of the control enzyme in the sample, a new aliquot of the sample was analyzed. Enzyme activity was defined as the amount of product of the reaction, in units of μmol/L/h. In accordance with previously published recommendations (Savostyanov et al., 2021), the cutoff for GAA activity was chosen as 2.15 μmol/L/h.

### 2.3 DNA isolation

Genomic DNA was extracted from dried blood spots (DBS) with the MagPure Universal DNA Kit (Magen, Guangzhou, China) using the automated Auto-Pure 96 DNA isolation station (Allsheng, Hangzhou, China). DNA quality and concentration were measured either by spectrophotometry with the NanoPhotometer N60 (Implen Gmbh, Munich, Germany), or with the Qubit dsDNA HS Assay Kit and Qubit 3.0 fluorometer (Invitrogen, Waltham, MA, United States).

### 2.4 Sanger sequencing

All primers used for *GAA* (*NM_000152.5*) sequencing were designed with “Beacon Designer 8.10”(PREMIER Biosoft International), and their specificity was confirmed using “Primer-BLAST.” Oligonucleotides were synthesized by JSC “Evrogen.” DNA amplification took place on Bio-Rad T100 (Bio-Rad, United States) and ProFlex (Thermo Fisher Scientific, Waltham, MA, United States) thermocyclers. Sequencing reactions employed the BigDye® Terminator v3.1 Cycle Sequencing Kit (Thermo Fisher Scientific, Waltham, MA, United States) following the manufacturer’s protocol. Capillary electrophoresis was conducted on ABI 3500XL and ABI 3500 genetic analyzers (Thermo Fisher Scientific, Waltham, MA, United States). The obtained sequences were compared against RefSeqGene references from the National Center for Biotechnology Information (NCBI) database.

### 2.5 MLPA analysis

Multiplex Ligation-Dependent Probe Amplification (MLPA) method was applied to detect deletions and duplications using the SALSA MLPA Probemix P453 (MRC Holland, Amsterdam, Netherlands) as per manufacturer’s instructions. Denaturation, hybridization, ligation and amplification were performed on Thermocyclers T100 (Bio-Rad, Hercules, CA, United States) and ProFlex (Thermo Fisher Scientific, Waltham, MA, United States), and capillary electrophoresis was performed on ABI 3500 (Thermo Fisher Scientific, Waltham, MA, United States) genetic analyzer.

### 2.6 Next-Generation Sequencing (NGS)

The target regions of the *ANO5*, *CAPN3*, *DYSF*, *FKRP*, *GAA*, *SGCA*, *SGCB*, *SGCD*, *SGCG*, and *TCAP* genes were analyzed. Hybridization probes for the NGS panel were created with the Hyper Design Tool (Roche Diagnostics, Indianapolis, IN, United States) to cover all coding and splice regions of the listed genes. Library preparation followed the KAPA HyperPlus Kit protocol, using a 15-min DNA fragmentation to yield ∼350 bp fragments. Target enrichment used KAPA HyperCap probes (Roche), and sequencing was performed on the MiSeq platform (Illumina Inc., San Diego, United States) with V2 chemistry (500 cycles, paired-end). Each run generated an average of 31.5 million reads, 88% above Q30, with 99% of target regions covered at least 15 times and a mean read depth of 100x.

### 2.7 Bioinformatic analysis

Bioinformatic analysis was carried out according to the guidelines of Genome Analysis Toolkit Best Practices (Broad Institute GATK, 2025). Briefly, raw reads were trimmed using Trimmomatic (version 0.39) and sequence alignment was performed with Burrows–Wheeler Aligner (version 0.7.17) using GRCh38 genome assembly as a reference. Next, duplicate reads were marked using Picard tools, and base quality score recalibration (BQSR) was performed. Then, genetic variations (SNPs and indels) were identified with GATK HaplotypeCaller (version 4.1.2). Next, gene annotation was performed with an in-house script to annotate variations present in ClinVar, OMIM, and HGMD databases.

All sequence variants, obtained from Sanger sequencing and NGS with minor allele frequency of <1% according to the Genome Aggregation Database (gnomAD) (available at: https://gnomad.broadinstitute.org), were subjected to bioinformatics analysis in the program “Alamut Visual” (Interactive Biosoftware). *In-silico* tools SIFT, PolyPhen HDIV, PolyPhen HVAR, Mutation Taster, FATHMM, CADD13, DANN, M-CAP and REVEL, as well as Alamut with the built-in software modules were used for the prediction of the pathogenicity of the missense sequence variants. The guidelines of the American College of Medical Genetics and Genomics (ACMG) manual were used for the variants interpretation (Richards et al., 2015).

### 2.8 Statistical analysis

Statistical analysis was performed using the “Statistica 10.0” software package (StatSoft). For quantitative data use The Mann–Whitney p-test was used. The ROC method was used to determine the optimal cutting point corresponding to the maximum sensitivity and specificity of the method. The SPSS software and R were also used for statistical analysis. Calculation of reference intervals was performed following the previously published recommendations (Horn and Pesce, 2003).

## 3 Results

### 3.1 The study cohort and diagnostic algorithms

**FIGURE 1:**
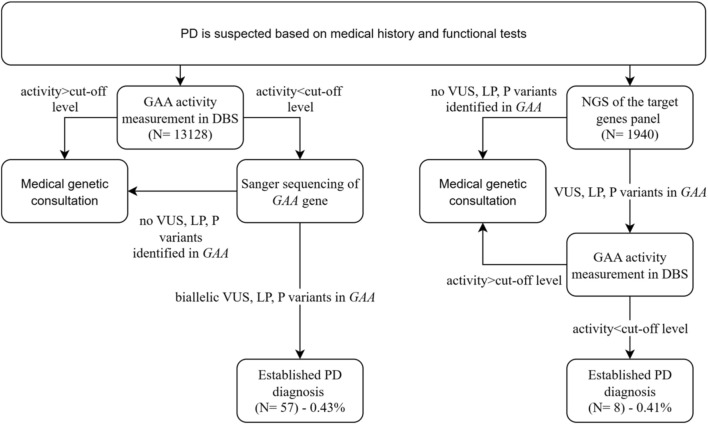
The flowchart of screening algorithms and outcomes. VUS, variant of uncertain significancy, LP, likely pathogenic variant, P, pathogenic variant.

Here, we performed molecular screening in a cohort of 15,068 participants with suspected PD of a wide range of age from several regions of the Russian Federation. Two screenings had been undertaken from 2014 to 2025, and 2021 to 2025, respectively: 13,128 patients were screened using “two-tier one-gene” algorithm (measurement of GAA activity in DBS followed by Sanger sequencing of the *GAA*) and 1940 patients were screened using NGS-based algorithm followed by measurement of GAA activity to confirm diagnosis. The study design and screening outcomes are depicted in Figure 1.

**FIGURE 2:**
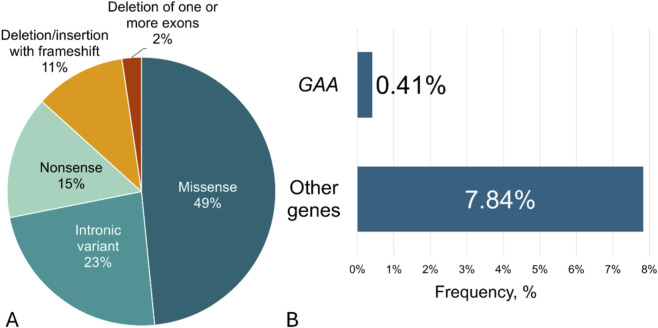
**(A)** The total frequencies and type of nucleotide variants found in *GAA* gene using both algorithms in a cohort of 15,068 participants; **(B)** The frequencies of causal nucleotide variants found in *GAA* gene or other genes using NGS in a cohort of 1940 participants.

Several nucleotide variants within the *GAA* gene (NM_000152.5) and other genes from the NGS panel were identified in our study (Supplementary Table S1). Causative nucleotide variants in the *GAA* gene were found in 0.41% of cases, and in other genes from the NGS panel in 7.8% (Figure 2). Using “two-tier one-gene” algorithm, causative nucleotide variants in *GAA* gene were found in 0.47% of the cases.

**FIGURE 3:**
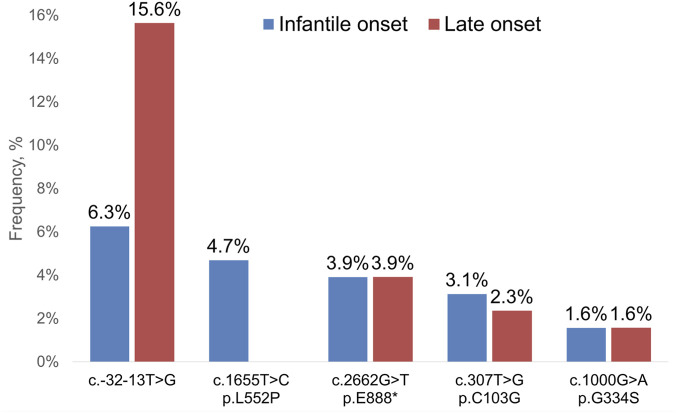
The frequencies and spectrum of the most common sequence variants found in *GAA* gene using both algorithms in a cohort of 15,068 participants.

The frequencies and spectrum of the most common nucleotide variants found in the *GAA* gene using both algorithms in sub-cohorts of patients with IOPD and LOPD are shown in Figure 3.

### 3.2 NGS-based screening

**FIGURE 4:**
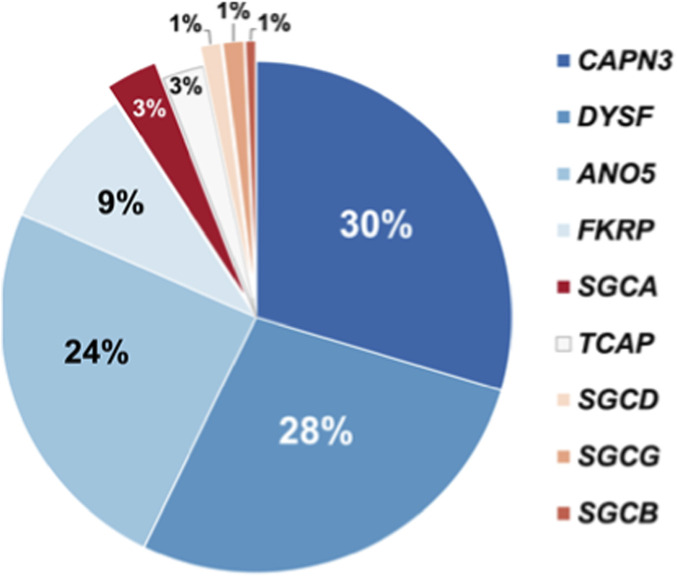
Pie-chart depicting percentage of causal nucleotide variants identified by NGS across the panel of the genes.

1,940 patients were screened using an NGS-based algorithm. As a result of screening total of 138 patients (7.1%) were diagnosed with various muscular dystrophies, myopathies, or PD. The majority of these individuals had biallelic variants in the *CAPN3* gene (30%), indicative of calpainopathy, 28% of patients had biallelic variants in gene *DYSF,* indicative of dysferlinopathy, 24% of patients had biallelic variants in gene *ANO5*, indicative of muscular dystrophy, and in 9% of patients biallelic variants were found in gene *FKRP* (diagnosed with dystroglycanopathy). In 3% of cases biallelic variants were found in gene *SGCA* (diagnosed with α-sarcoglycanopathy). In 3% of patients biallelic variants were found in gene *TCAP* (diagnosied with limb-girdle muscular dystrophy type 2G). In 1% of cases, genes *SGCB, SGCD, SGCG* had biallelic variants, indicative of beta-sarcoglycanopathy, delta-sarcoglycanopathy, and gamma-sarcoglycanopathy, correspondingly. The percentage of causal nucleotide variants identified by NGS across the panel of the genes is presented in Figure 4.

In 20 patients (1.4%) harboring biallelic variants in the *GAA* gene, GAA enzyme activity was evaluated by High-Performance Liquid Chromatography (HPLC)/Tandem Mass Spectrometry (HPLC-MS/MS), resulting in the identification of 8 (0.41%) patients with PD. Additionally, at least one pseudodeficiency allele was detected in 190 patients (9.8%): rs1800307 (c.1726G>A, p. G576S) was found in 1.3% alleles, and rs1800309 (c.2065G>A, p. G689K) in 5.1% alleles, among others.

### 3.3 “Two-tier one-gene” screening

Total 13,128 patients were screened using “two-tier one-gene” algorithm. The HPLC-MS/MS identified 111 patients exhibiting enzyme activity values below the established cut-off (2.15 μmol/l/h), these patients underwent Sanger sequencing of the *GAA* gene. Among them, 54 patients were identified with biallelic VUS, LP, or P variants. Other 57 patients did not have biallelic variants in the *GAA*, and, within this group, 32 carried at least one pseudo-deficiency allele (rs1800307, c.1726G>A or rs1800309, c.2065G>A).

### 3.4 Genetic findings

**FIGURE 5:**
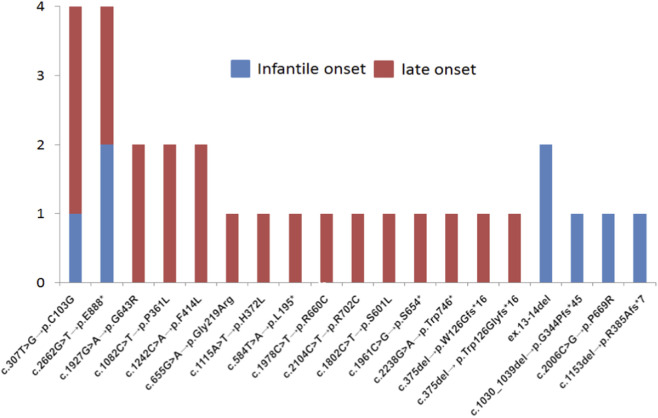
The numbers and spectrum of nucleotide variants found in compound heterozygous state with c.-32-13T>G.

In our cohort, all patients with c.-32-13T>G variant were compound heterozygous. The numbers and spectrum of nucleotide variants found in compound heterozygous state with c.-32-13T>G are shown in Figure 5.

**FIGURE 6:**
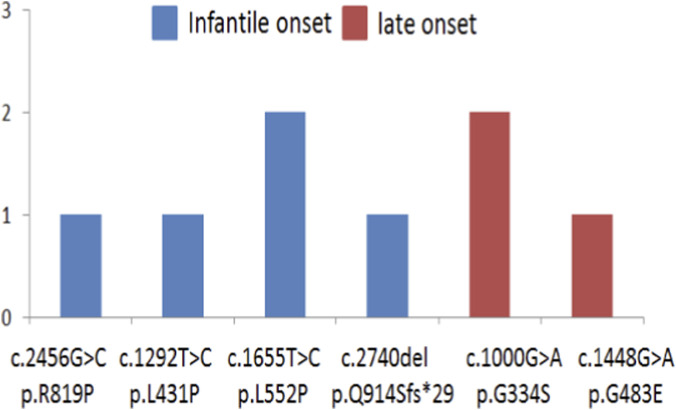
The numbers and spectrum of nucleotide variants found in homozygous state.

The majority of other causal nucleotide variants were also in compound heterozygous state, however several patients harbored such variants in homozygous state. In particular, 2 patients were homozygous for c.1000 G>A, p. G334S, 2 patients were homozygous for c.1655T>C, p. L552P. One was homozygous for c.2456G>C, p. R819P; one for c.1292T>C, p. L431P; one for c.1448G>A, p. G483E; one for c.2740del, p. Q914Sfs*29 (Figure 6).

Total 25 novel nucleotide variants in *GAA* were described (Supplementary Table S2).

### 3.5 Genotype-phenotype correlations

Several differences in clinical signs were found for IOPD vs. LOPD patients (Table 1).

**TABLE 1:** Clinical signs in patients with IOPD and LOPD.

Clinical sign	IOPD	LOPD	p-value (chi-square)
Hepatomegaly	11	4	<0.001
No hepatomegaly	4	12	
Left ventricular hypertrophy	14	2	0.001
No left ventricular hypertrophy	3	19	
Heart rhythm disturbances	12	5	0.012
No heart rhythm disturbances	5	16	

The analysis also revealed that LOPD patients were statistically significantly more likely (by a factor of 2.96) to possess a genotype with one allele being c.-32-13T>G. Consequently, we elected to conduct a genotype-phenotype correlation study comparing two groups: patients with exclusively biallelic missense variants (Group 1) and with biallelic variants, one of which is c.-32-13T>G (Group 2). Following genotype-phenotype correlations were found (Table 2).

**TABLE 2:** Clinical signs in patients with different types of nucleotide variants in the *GAA* gene: genotype-phenotype correlation.

Clinical sign	Group I ^a^	Group II ^b^	p-value (chi-square)
Heart rhythm disturbances	7	4	0.006
No heart rhythm disturbances	1	12	
Motor development delay	7	1	<0.001
No motor development delay	2	17	
Hepatomegaly	6	3	0.012
No hepatomegaly	3	16	
Left ventricular hypertrophy	5	2	0.020
No left ventricular hypertrophy	4	17	

^a^ Patients only with biallelic missense variants.

^b^ Patients with biallelic variants, one of which is c.-32-13T>G.

Furthermore, logistic regression analysis was conducted to assess the likelihood of clinical manifestations relative to the subtype of Pompe disease (IOPD or LOPD), the classification of nucleotide variants identified (Group1 or Group2), and enzyme activity levels. The results indicated a statistically significant increase in the probability (OR = 1.48) of delayed motor development in IOPD patients possessing biallelic missense variants (Group 1). No association was observed between clinical manifestations and enzyme activity.

## 4 Discussion

The rapid progress in the sequencing techniques in the last decade made it feasible to use NGS as a first-tier diagnostic tool in a clinical setting; however, Sanger sequencing still remains a gold standard in diagnostics. Commonly used approach to establish a PD diagnosis combines an initial enzyme assay (EA) measuring GAA activity (usually, in DBS) (Bodamer et al., 2017) and subsequent *GAA* genotyping (usually, via Sanger sequencing) in case of decreased enzymatic activity of GAA and suspicion of PD based on the overall clinical picture. Such EA is commonly performed using fluorometry-based analysis or tandem mass spectrometry, the latter allowing simultaneous measurement of activities of several enzymes. For example, as early as in 2005, a pilot newborns screening (NS) for PD was started at the National Taiwan University Hospital using a fluorometric EA. Of the total 206,088 screened infants, 6 were diagnosed with PD (Chien et al., 2009).

Notably, there are several limitations of EA assays and several issues to consider. The affinity GAA to glycogen is much higher compared to “artificial” substrate (4-methylumbelliferyl-α-D-glucopyranoside, 4-MU), commonly used in EA (Wisselaar et al., 1993). There is a risk of getting false negative results. GAA activity overlaps with activity of other enzymes, namely, “leukocyte” neutral glucosidase II (GANAB), neutral α-glucosidase C (GANC), and maltase-glucoamylase (MGAM) which also use 4-MU as a substrate (Lukacs et al., 2020). GANAB and GANC do not have any significant activity in acid environments and do not interfere with assay, while MGAM is a main contributor to the false positive results in case of DBS sample analysis. Thus, usage of inhibitors of MGAM activity, maltose or acarbose, is required to minimize its interference with the fluorometric assay (Zhang et al., 2006). The activity of reference enzymes (RE) should be measured in parallel with GAA to monitor sample quality (Lukacs et al., 2020). The pre-analytical factors, such as specimen collection (an effect of drying conditions in case of DBS), its storage, transportation, and others, might affect the sensitivity of the test.

Here, we used HPLC-MS/MS method for GAA activity measurement which was previously optimized and comprehensively characterized by our team in a smaller scale pilot PD screening (Savostyanov et al., 2021).

Currently, PD is not included in any nation-wide NS, anywhere. However, in the US, it was included in the recommended uniform screening panel (RUSP) in February 2015. As an example of the implementation of such recommendation, in Pennsylvania detection rate of 1/16,095 (IOPD + LOPD) was reported based on results of the screening of 531,139 newborns performed between February 2016 and December 2019 (Ficicioglu et al., 2020). The study findings also included 15 newborns with one or two pseudodeficiency alleles and 2 false positive newborns. The screening was done using flow-injection tandem mass spectrometry (FIA/MS/MS) and full sequencing of *GAA*.

Our study is one of the few selective screenings for PD performed in the world. Results of selective screenings of patients with suspected PD, including adults with suspected LOPD, have been previously reported ((Meznaric et al., 2019; Sekulic et al., 2025) and others). As an example, one of the targeted NGS gene panel for differential diagnostics of PD included, alongside *GAA,* nine genes linked to diseases from a group of autosomal recessive limb–girdle muscular dystrophies (*CAPN3*, *DYSF, SGCA*, *SGCB*, *SGCD*, SGCG, *FKRP*, *ANO5*, *TCAP)* (Bevilacqua et al., 2024; Ficicioglu et al., 2020), the same as in our panel. Use of this panel in a 21-country multicentric analysis of 2,372 patients with progressive limb–girdle muscular weakness allowed diagnosing 86.2% and 13.8% of patients with LGMD and PD, correspondingly (Bevilacqua et al., 2024). Causative nucleotide variants (P, LP) were detected in 11% of patients compared to 8.5% in our cohort, perhaps due to population differences. Similarly to our study, the greatest numbers of causative nucleotide variants were detected in *CAPN3* and *DYSF* genes (in 23.37% and 26.44% of cases, respectively) (Bevilacqua et al., 2024). The NGS panel used in Argentina on a cohort of 472 patients with muscle weakness included genes *SGCA, SGCB, SGCG, SGCD, CAPN3, DYSF, TCAP, FKRP, ANO5, HNRPDL, GAA, CAV3*. The most frequently causative nucleotide variants were detected in *CAPN3* gene. The NGS of the panel was carried out for screening purposes in 0.4% of patients with definitive diagnosis of PD (Schiava et al., 2022).

In the case of PD, many causal nucleotide variants are “private”, i.e., found only in one family or in a very small population. Other PD-causing nucleotide variants are more common, and some of the common variants are more frequent in particular populations. For example, the “classic” nucleotide variant c.-32-13T>G (also known as IVS1) in the splicing site upstream of the 2nd exon of *GAA* is the most common among LOPD individuals in the European population (Niño et al., 2019). The allelic frequencies of this variant in PD patients range between 40% and 70% in some European populations (reviewed in (Peruzzo et al., 2019)). This variant is known to cause impaired splicing and production of three different “abnormal” splicing variants. It is considered that c.-32-13T>G affects overall splicing efficiency of the *GAA* pre-mRNA transcript, but some residual “normally spliced’ *GAA* mRNA is present in the cells with c.-32-13T>G, resulting in the delayed disease manifestation (reviewed in (Dardis et al., 2014)). It has been shown that c.510C>T variant is a modifier of the PD phenotype in compound heterozygous and homozygous c.-32-13T>G patients, as presence of c.510C>T decreases the levels of “normally spliced” *GAA* mRNA in the cell. Thus, compound heterozygous c.-32-13T>G patients with c.510C>T variant might have a childhood onset of PD (Bergsma et al., 2019). Other variants frequently found in compound heterozygous state with c.-32-13T>G are c.525del, c.2481 + 102_2646 + 31del; c.1927G>A (comprehensively reviewed in (Taverna et al., 2020)). Surprisingly, homozygosity of c.-32-13T>G is an unexpectedly rare event, so far being observed only in a few cases; moreover, c.-32-13T>G causal variant has never been observed in a homozygous state in classic IOPD, whereas in LOPD its homozygosity has been reported (Musumeci et al., 2015).

In our study it was also the most frequent nucleotide variant in the *GAA* gene, and, in line with previous publications, found exclusively in compound heterozygous state. Previously, it been shown that the combination of any splice site variant from the following, c.546G>C, c.1076–22T>G, c.2646 + 2 T>A, with c.−32–13T > G is more likely to be found in patients with LOPD (Viamonte et al., 2021).

In our study, variants most commonly found in compound heterozygous state with c.-32-13T>G were (in both IOPD and LOPD) c.307T>G, c.2662G>T, followed by c.1927G>A, c.1082C>T, c.3175del and (in IOPD) ex.13-14del (Figure 5). Finally, in our cohort variant c.-32-13T>G was more common in patients with LOPD (15.4%) compared to IOPD (6.2%), confirming previously reported findings from other populations.

The second most common causal variant in our cohort was c.2662G>T, p.E888*, detected in 3,8% of IOPD and 3,8% LOPD (Figure 5). Interestingly, the c.2662G>T is relatively common in Asian populations. For example, its allelic frequency in northern and southern Chinese patients with IOPD has been previously reported as 23.1% and 4.2%, respectively (Chen et al., 2017).

In our cohort variant c.1655T>C, p.L552P was relatively common among patients with IOPD (4,6%) but absent in LOPD. Such finding is rather unexpected given that it is a missense variant, and its presence does not result in a truncated protein. Notably, according to literature, this variant had previously been found in a patient with LOPD and is not exclusively IOPD-related (Joshi et al., 2008).

Some variants in *GAA* cause pseudodeficiency, such as a c.271G>A p. D91N (found in Caucasian populations) (Martiniuk et al., 1990), and c. [1726G>A; 2065G>A] (commonly found in Asian populations) (Tajima et al., 2007). Variant c.1726G>A, p. G576S in cis with c.2065G>A (so-called c. [1726A; 2065A] allele) is known to cause pseudodeficiency and is relatively common in Asian populations (Oda et al., 2011). Further, the c. [1726A; 2065A] homozygosity has been described in apparently healthy individuals in Japanese population (Kumamoto et al., 2009). It is also common among various Chinese populations (gnomAD 4.1: total 0.83%, East Asia 16.7%). Intriguingly, in our study, c.1726G>A was also found in 1.3% alleles, and c.2065G>A in 5.1% alleles (gnomAD 4.1: total 4.55%, East Asia 25.5%).

In light of the findings from the work by De Filippi et al. (De Filippi et al., 2010; De Filippi et al., 2014) [52–53], it’s plausible to suggest that the clinical phenotype of PD can be modulated by other genetic factors, such as sequence variants (including frequent polymorphisms) in the genes linked to other neuromuscular diseases and muscle tissue homeostasis. In such a scenario, of particular value might be the data obtained from NGS of the focused panel of “neuromuscular” genes, combined with the data from longitudinal study and allowing searching for possible additional modulators of PD phenotype.

Following genotype-phenotype correlations were found in our cohort: patients with biallelic missense variants compared to patients with biallelic variants, one of which is c.-32-13T>G, more often had heart rhythm disturbances (0,006), motor development delay (p < 0,001), hepatomegaly (0,012) and left ventricular hypertrophy (0,020). Notably, some missense variants found in our study and contributing to these genotype-phenotype correlations were also found in other studies in patients with IOPD. For example, position 643 is in the catalytic domain of GAA enzyme is close to its active site, and variant c.1927G>A, p. G643R, detected in our study, was previously reported as found in homozygous state IOPD patient from Tunisia (Alila-Fersi et al., 2020). Moreover, region including positions 638–645 is involved in the transport and maturation of the GAA protein (Li et al., 2023), significantly affecting its functions. In our cohort, missense variants c.1933G>A, p. D645N and c.1933G>C, p. D645H from this region were also present. Variant c.1933G>A, p. D645N was previously found in a homozygous state in IOPD patient from China (Kroos et al., 2012), indirectly confirming the impact of variants in this region on GAA function. Further, missense variants c.2456G>C p. R819P, c.1802C>T p. S601L, and c.1781G>A, p. R594H according to literature were predicted to cause severe phenotype of PD (Marotto et al., 2023). As for the frameshift and nonsense variants found in our study, in most cases they were found in the last exons of the gene, therefore only a relatively small part of the GAA protein close to the C-terminus was lost as a result.

In the current study, we also found that numbers of patients with such clinical signs as hepatomegaly, left ventricular hypertrophy and heart rhythm disturbances were statistically significantly higher in patients with IOPD compared to LOPD (Table 2), which is consistent with the current views on the pathogenesis of PD.

Of note, additional assessments of metabolic alterations and biomarkers associated with PD can also being used to aid diagnostics. For example, LOPD is often associated with elevated levels of serum Creatine kinase (CK), although some patients with LOPD might have levels of CK in the normal range (Marotto et al., 2023). Furthermore, measurement of glycogen-derived tetrasaccharides (TGLC) secreted in urine, namely glucose tetrasaccharide (Glc4), either alone or in combination with its isomer maltotetraose (M4) (Heiner-Fokkema et al., 2020), can also be used as a complementary method for PD diagnostic and monitoring. Notably, Glc4 is not a PD-specific biomarker and can be used for a number of other glycogen storage diseases (GSDs), such as GSDIa and GSDIb (OMIM#232200, also known as von Gierke disease and G6P transport defect, correspondingly), GSDIIIa and GSDIIIb (OMIM#232400, also known as Cori disease and Forbes disease, correspondingly), GSDXI (OMIM#612933, also known as Lactate dehydrogenase deficiency) (Heiner-Fokkema et al., 2020). M4 has not been studied much due to its rapid degradation in urine (the issue that can be mitigated by adjusting specimen’s pH to ∼9.5), but recent work by Ren et al. has demonstrated its utility as a PD biomarker (perhaps also not exclusively PD-specific) (Ren et al., 2023). Obviously, other complementary metabolic biomarkers yet to be established in the clinic (for example, L-phenylalanine, N-acetyl-4-aminobutanal, N-acetyl-L-aspartic acid, and others) might increase diagnostic accuracy (de Moraes et al., 2023).

In our study we designed and applied two diagnostic algorithms for selective PD screening (namely, “two tier one gene” based on Sanger sequencing or NGS-based one) and presented its results. In our study, causative nucleotide variants in *GAA* gene were found in 0.47% and 0,41% of cases using “two-tier one-gene” algorithm and NGS-based approach. A molecular diagnostics laboratory should take into account a time/cost balance when choosing the preferable algorithm of screening for PD. Undoubtedly the NGS-based algorithm is faster and provides more data, thus shortening the patient’s “diagnostic journey”. In particular, it allows establishing differential diagnostics with a number of other disorders, as it has been demonstrated in our study where significant percent of patients from the cohort (7.1%) were diagnosed with various muscular dystrophies and myopathies. One or the other algorithm can be chosen depending on the capabilities of the diagnostic center and the aims of the screening.

Overall, the current study is the first one that provides a comprehensive picture of the genetic features of the PD (both IOPD and LOPD) in Russia.

## Data Availability

The original contributions presented in the study are publicly available. This data can be found here: https://www.ncbi.nlm.nih.gov/bioproject/PRJNA1406018.

## Glossary

ACMG: American College of Medical Genetics and Genomics
ADA: anti-drug antibodies
BQSR: base quality score recalibration
CK: Creatine kinase
CRIM: Cross-reacting immunologic material
DBS: Dried blood spots
EA: enzyme assay
ERT: Enzyme replacement therapy
FIA/MS/MS: Flow-injection tandem mass spectrometry
GANAB: “leukocyte” neutral glucosidase II
GANC: neutral α-glucosidase C
GAA: Acid alpha-glucosidase
Glc4: Glucose tetrasaccharide
GSDII: Glycogen Storage Disease II
GSDs: glycogen storage diseases
HPLC-MS/MS: High-Performance Liquid Chromatography (HPLC)/Tandem Mass Spectrometry
HSAT: High-sustained rhGAA IgG antibody titers
IOPD: infantile-onset Pompe disease
LOPD: Late Onset Pompe Disease
LP: Likely pathogenic
MGAM: Maltase-glucoamylase
MLPA: Multiplex Ligation-Dependent Probe Amplification
MS/MS: tandem mass spectrometry
NCBI: National Center for Biotechnology Information
NGS: Next-Generation Sequencing
NS: newborn screening
NVs: nucleotide variants
PCT: pharmacological chaperone therapy
P: pathogenic
PD: Pompe disease
RE: reference enzymes
rhGAA: recombinant human GAA
RUSP: recommended uniform screening panel
TGLC: glycogen-derived tetrasaccharides
VUS: variant of uncertain significance
